# A Marine-Derived Sterol, Ergosterol, Mitigates UVB-Induced Skin Photodamage via Dual Inhibition of NF-κB and MAPK Signaling

**DOI:** 10.3390/md23110445

**Published:** 2025-11-19

**Authors:** Junming Zhang, Jiangming Zhong, Yi Li, Qi Zhou, Zhiyun Du, Li Lin, Peng Shu, Ling Jiang, Wei Zhou

**Affiliations:** 1 HBN Research Institute and Biological Laboratory, Shenzhen Hujia Technology Co., Ltd., Shenzhen 518000, China; 2 School of Biomedical and Pharmaceutical Sciences, Guangdong University of Technology, Guangzhou 510006, China; 3 Foshan Allan Conney Biotechnology Co., Ltd., Foshan 523281, China

**Keywords:** ergosterol, UVB radiation, photoaging, skin photodamage, inflammation, redox signaling

## Abstract

Background: Ultraviolet B (UVB) radiation induces oxidative stress, inflammation, and collagen degradation in skin, leading to photodamage. Ergosterol (ERG)—a sterol widely distributed in fungi and algae, including numerous marine species—possesses antioxidant and anti-inflammatory activities, but its photoprotective mechanisms remain unclear. Methods: Using integrated *in vitro* (UVB-irradiated human keratinocytes) and *in vivo* (topical ERG in a murine UVB model) approaches, combined with transcriptomic and network pharmacology analyses, we evaluated ERG’s effects on oxidative stress, inflammation, and extracellular matrix integrity. Results: ERG treatment preserved keratinocyte viability, reduced reactive oxygen species, and suppressed pro-inflammatory mediators after UVB exposure. In mice, topical ERG significantly attenuated epidermal hyperplasia, maintained tight-junction integrity, and inhibited collagen matrix degradation. Mechanistically, ERG exerted dual inhibition of the nuclear factor kappa B (NF-κB) pathway, which mediates inflammation, and the mitogen-activated protein kinase (MAPK) pathway, which regulates collagen degradation. Conclusions: These findings identify ERG as a marine-derived sterol with potent photoprotective activity that simultaneously targets oxidative stress, inflammation, and extracellular matrix damage, highlighting its promise as a natural compound for dermatological applications and aligning with ongoing efforts to explore marine-derived agents against skin oxidative stress and inflammation.

## 1. Introduction

The skin is the largest organ of the human body, serving as a crucial protective barrier between the internal system and external environments. Its multi-layered structure, consisting of the epidermis, dermis, and subcutaneous tissue, allows it to perform important functions, including defending against ultraviolet rays, regulating body temperature, and maintaining water balance [1,2,3].

Ultraviolet B (UVB) radiation is a major extrinsic factor driving skin aging and photodamage, primarily through induction of reactive oxygen species (ROS) and reactive nitrogen species (RNS). Long-term exposure to UVB initiates a cascade of detrimental effects, including severe skin oxidative stress, inflammation, collagen degradation, and cell apoptosis, with clinical manifestations including wrinkles, skin redness and laxity, uneven pigmentation, and even skin cancer [4,5,6]. Excessive ROS production initiates oxidative stress–mediated signaling cascades, activating transcription factors such as nuclear factor kappa B (NF-κB) and activator protein 1 (AP-1) via the mitogen-activated protein kinase (MAPK) pathway. These pathways coordinate inflammatory mediator release, such as interleukin-1β (IL-1β), interleukin-6 (IL-6), and tumor necrosis factor-α (TNF-α), and extracellular matrix degradation through matrix metalloproteinases (MMPs), and impairment of skin barrier function [7,8,9]. RNS primarily generate nitric oxide (NO), which acts synergistically with ROS to induce inflammatory responses and apoptosis, thereby exacerbating skin photodamage [10]. Furthermore, UVB directly penetrates the epidermis, damages cellular DNA, and forms cyclobutane pyrimidine dimers (CPDs), which if unrepaired, may lead to the accumulation of mutations and promote the occurrence of skin cancer [11,12,13]. Together, these redox-sensitive processes contribute to epidermal thickening; collagen fragmentation; barrier dysfunction; and, ultimately, photoaging.

A variety of small molecules and natural products have been investigated for their capacity to mitigate UVB-induced oxidative stress. Classical antioxidants, including vitamin E (VE), primarily function as ROS scavengers, but often show limited efficacy in suppressing downstream redox-sensitive signaling events. Additionally, even a proven anti-photoaging agent like vitamin A (also known as retinol), which modulates gene expression to repair photoaged skin, may cause significant skin irritation [14]. Thus, the identification of compounds that act beyond direct ROS neutralization and modulate redox signaling networks is of significant interest in the field of redox biology and skin aging.

Ergosterol (ERG) is a natural steroid compound broadly distributed across fungi and algae, including numerous marine species such as marine fungi and macroalgae [15]. In marine ecosystems, ERG serves as a major sterol and is widely used as a biomarker for fungal biomass, having been directly isolated from marine-derived fungi [15], microalgae [16], and sponges [17]. Beyond its terrestrial occurrence, this marine-derived sterol exhibits multiple pharmacological activities, including anti-inflammation, antibacterial, antioxidant, and anti-tumor properties [18,19,20]. Structurally, the presence of conjugated double bonds in ERG is critical for its antioxidant capacity, as studies have demonstrated that ERG protects lipids against oxidation through an electron transfer followed by proton transfer (ET-PT) mechanism [21]. This redox activity is attributed to the enhanced stability of the radical cation intermediate due to electron delocalization across the conjugated system, thereby enabling ERG to effectively scavenge free radicals and mitigate oxidative damage. Critically, ERG is the biological precursor to vitamin D2, a molecule whose metabolism is intrinsically linked to skin physiology and its response to UV radiation [22,23]. However, its potential role as a redox-active compound in skin biology remains largely unexplored. Given its structural features and bioactivity, ERG may act not only by limiting ROS accumulation but also by directly influencing redox-sensitive pathways implicated in skin inflammation and extracellular matrix degradation.

Previous research in other biological systems provides a strong rationale for investigating ERG in the context of skin. ERG was shown to significantly inhibit lipopolysaccharide (LPS)-induced microglial cell activation and neuroinflammatory responses by inhibiting the NF-κB, protein kinase B (AKT), and MAPK signaling pathways, reducing the release of pro-inflammatory cytokines such as IL-1β and TNF-α—the same pathways central to UVB-induced inflammation in the skin [24,25]. Similarly, ERG was found to mitigate oxidative stress-induced cell damage by enhancing the antioxidative response and promoting the clearance of ROS [26]. Given that oxidative stress and inflammation are the primary drivers of skin photodamage, these studies strongly suggest that ERG possesses the necessary mechanism to counteract the effects of UVB and bear the potential to intervene in UVB-induced skin photodamage [27].

In this study, we investigated the protective effects of ERG against UVB-induced oxidative stress and skin photodamage using *in vitro* keratinocyte and macrophage models, an *in vivo* murine UVB irradiation model, and integrative transcriptomic and network pharmacology analyses. We show that ERG suppresses ROS accumulation, inhibits NF-κB and ERK activation, reduces inflammatory mediator release, preserves tight junction integrity, and prevents collagen degradation. Our findings establish ERG as a novel redox-active sterol with dual actions on oxidative stress and redox signaling pathways, highlighting its potential relevance to oxidative-stress-driven skin aging and photodamage.

## 2. Results

### 2.1. ERG Alleviates UVB-Induced Photodamage in HaCaT Cells

**Figure 1:**
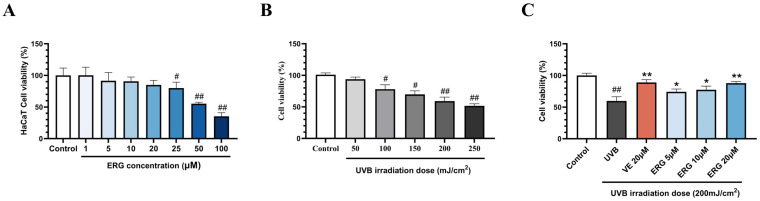
Ergosterol (ERG) protected HaCaT cells from ultraviolet B (UVB)-induced cytotoxicity. (**A**) Cell viability of HaCaT cells following treatment with increasing concentrations of ERG to assess its intrinsic cytotoxicity. (**B**) Dose-dependent effect of UVB irradiation on HaCaT cell viability, used to establish the optimal damage model dosage. (**C**) Protective effects of ERG (5, 10, 20 μM) and Vitamin E (VE, 20 μM) on the viability of HaCaT cells exposed to UVB radiation (200 mJ/cm^2^). Data are presented as the mean ± SD of three independent experiments (*n* = 3). # *p* < 0.05 and ## *p* < 0.01 indicate significant differences compared to the Control group. * *p* < 0.05 and ** *p* < 0.01 indicate significant differences compared to the UVB Model group.

To establish optimal experimental parameters, we first determined the non-toxic concentrations of ERG and an effective damage-inducing dose of UVB in HaCaT cells. Concentration of ERG up to 20 μM did not significantly impact cell viability of HaCaT cells (viability > 85%, Figure 1A). Based on this, three concentration gradients of ERG, 5, 10, and 20 μM were selected for further study. Additionally, HaCaT cells were irradiated with different doses of UVB (50–250 mJ/cm^2^) to assess its impact on cell activity (Figure 1B). Conversely, UVB irradiation decreased cell viability in a dose-dependent manner. A dose of 200 mJ/cm^2^, which reduced viability to approximately 60%, was chosen to model photodamage in subsequent experiments.

Next, we investigated ERG’s ability to protect HaCaT cells from UVB-induced cytocity. As shown in Figure 1C, UVB irradiation alone significantly reduced cell viability to 59.8 ± 5.3% compared with the control group. However, post-treatment with ERG led to a robust, concentration-dependent recovery, with viability increasing to 74.3 ± 3.5% (ERG, 5 μM), 77.5 ± 4.7% (ERG, 10μM), and 87.9 ± 1.9% (ERG 20 μM). Notably, the protective effect of high dose of ERG was comparable to that of 20 μM VE (88.9 ± 3.6%), demonstrating a significant restoration of cell viability of HaCaT cells.

### 2.2. ERG Mitigates UVB-Induced ROS in HaCaT Cells

Having established that ERG rescues HaCaT cells from UVB-induced cytotoxicity, we next investigated whether this was due to antioxidant activity. Since UVB radiation is known to exert its damaging effects primarily through the generation of excessive reactive oxygen species (ROS) via mitochondrial dysfunction and NADPH oxidase activation [28,29] and ERG was found to mitigate oxidative stress [26], we hypothesized that ERG protects cells by mitigating the generation of reactive oxygen species (ROS), a primary driver of photodamage.

**Figure 2:**
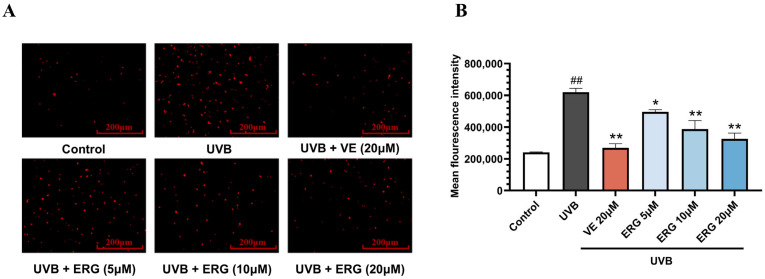
ERG effectively attenuates UVB-induced intracellular reactive oxygen species (ROS) accumulation in HaCaT cells. Cells were treated with ERG (5, 10, 20 μM) or VE (20 μM) 30 min after UVB exposure (200 mJ/cm^2^). Intracellular ROS levels were then assessed using the fluorescent probe DCFH-DA. (**A**) Representative fluorescence micrographs of DCFH-DA-stained cells, where the intensity of red fluorescence corresponds to the level of intracellular ROS. Scale bar: 200 μm. (**B**) Quantification of the relative mean fluorescence intensity across treatment groups. Data are presented as the mean ± SD of three independent experiments (*n* = 3). ## *p* < 0.01 indicates a significant difference compared to the Control group. * *p* < 0.05 and ** *p* < 0.01 indicate significant differences compared to the UVB Model group.

As expected, intracellular ROS levels in the HaCaT cells dramatically increased after exposure to UVB radiation (200 mJ/cm^2^) as illustrated with DCFH-DA fluorescence staining. Representative images in Figure 2A and quantitative analysis (Figure 2B) revealed that treatment with ERG following UVB exposure led to a significant, concentration-dependent suppression of ROS. VE exhibited potent efficacy in reducing ROS generation. Notably, highest concentration of ERG (20 μM) reduced ROS levels by approximately (inhibition rate, 47.2 ± 6.5%), an efficacy comparable to the positive control, VE (inhibition rate 56.7 ± 2.5%). These results demonstrate that ERG’s photoprotective effect is mediated, at least in part, by its ability to curb UVB-induced oxidative stress.

### 2.3. ERG Alleviates Inflammation in LPS-Induced RAW264.7 Cells

**Figure 3:**
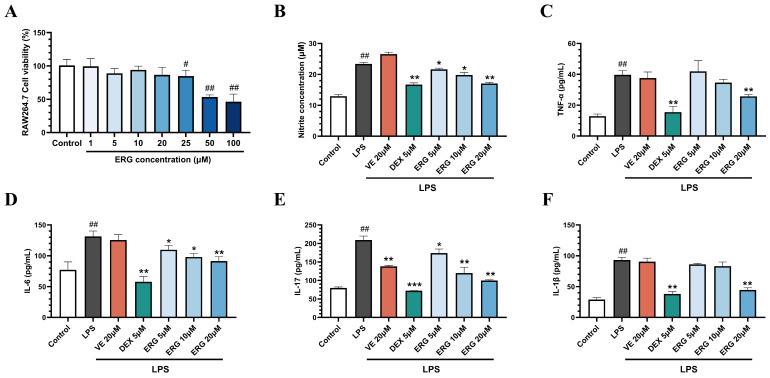
ERG attenuates the lipopolysaccharide (LPS)-induced inflammatory response in RAW264.7 macrophages. Cells were pre-treated with ERG (5, 10, 20 μM) or a positive control (dexamethasone, DEX) prior to stimulation with LPS (concentration 1 μg/mL). (**A**) Cell viability following ERG treatment, as determined by MTT assay. (**B**) Inhibitory effect of ERG on LPS-induced nitric oxide (NO) production, quantified using the Griess reagent. (**C**–**F**) Inhibitory effect of ERG on the secretion of pro-inflammatory cytokines: (**C**) Tumor necrosis factor-α (TNF-α), (**D**) Interleukin-6 (IL-6), (**E**) Interleukin-17 (IL-17), and (**F**) Interleukin-1β (IL-1β). Data are presented as the mean ± SD (*n* = 3). # *p* < 0.05 and ## *p* < 0.01 indicate significant differences compared to the untreated Control group. * *p* < 0.05, ** *p* < 0.01, and *** *p* < 0.001 indicate significant differences compared to the LPS-only Model group.

Beyond direct oxidative stress, a critical component of the UVB-induced damage cascade is a robust inflammatory response, which is orchestrated by immune cells such as macrophages [5]. To determine if ERG’s protective properties extend to modulating inflammation, we assessed its anti-inflammatory potential in a classic *in vitro* model of LPS-stimulated RAW264.7 macrophages. First, a preliminary MTT assay established that ERG did not exhibit significant cytotoxicity at concentrations of 5, 10, and 20 μM (cell viability > 85%); these concentrations were therefore selected for the subsequent experiments (Figure 3A).

Nitric oxide, a reactive molecule catalyzed by inducible nitric oxide synthase (iNOS), exacerbates inflammatory responses at high concentrations [30]. In LPS-stimulated macrophages, a significant increase in NO production was observed compared to the control group. Treatment with ERG dose-dependently suppressed this LPS-induced NO production. Notably, the inhibitory effect of the high-dose ERG (20 μM) group was comparable to that of the positive control, dexamethasone. In contrast, VE did not exhibit significant anti-inflammatory activity in LPS-only model (Figure 3B).

TNF-α, IL-17, IL-6, and IL-1β are key pro-inflammatory cytokines involved in the pathogenesis of inflammatory diseases. We then quantified the secretion of key pro-inflammatory cytokines. LPS induction markedly elevated the secretion of TNF-α, IL-6, IL-17, and IL-1β (Figure 3C–F). Treatment with ERG significantly attenuated the release of all four cytokines. Specifically, the ERG (20 μM) group reduced TNF-α and IL-6 levels by 34.98 ± 3.57% and 30.41 ± 1.40%, respectively. A more potent inhibitory effect was observed for IL-17 and IL-1β, with reductions of 52.10 ± 2.74% and 52.08 ± 1.62%, respectively, in the same treatment group. These results preliminarily indicate that ERG possesses anti-inflammatory activity.

### 2.4. ERG Alleviates UVB-Induced Epidermal Damage and Barrier Dysfunction in Mice

Our prior *in vitro* results demonstrated that ERG possesses both antioxidant and anti-inflammatory properties. To investigate whether these promising effects translate into therapeutic efficacy *in vivo*, we established a UVB-induced skin damage model in Balb/c mice.

**Figure 4:**
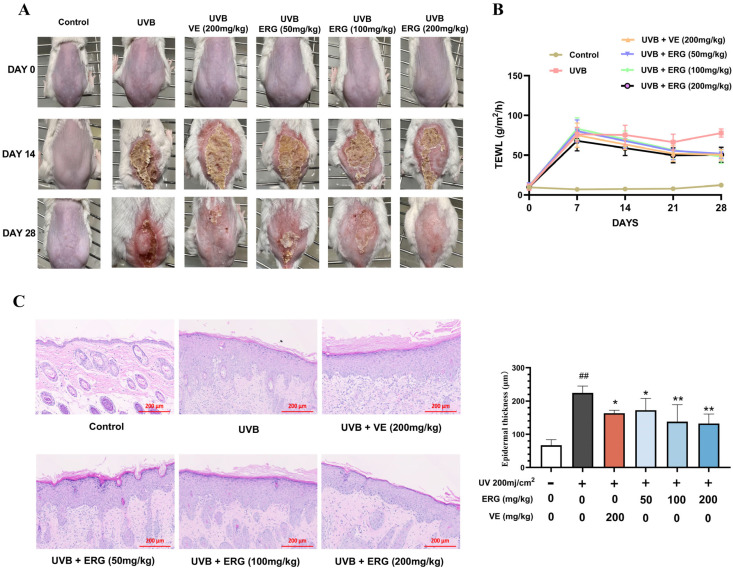
ERG alleviates UVB-induced (200 mJ/cm^2^ per day) epidermal damage in mouse skin. (**A**) Representative macroscopic views of the dorsal skin of mice. (**B**) Changes in trans-epidermal water loss (TEWL) of mouse skin over 28 days. (**C**) Epidermal thickness of mouse dorsal skin, as quantified from Hematoxylin-eosin (H&E) stained sections. Data are presented as the mean ± SD (*n* = 6 mice per group). ## *p* < 0.01 indicate significant differences compared to the untreated Control group. * *p* < 0.05 and ** *p* < 0.01 indicate significant differences compared to the UVB-only Model group.

Specifically, we exposed the dorsal skin of female Balb/c mice to UVB irradiation and topically applied either vehicle (propylene glycol, PEG 400) or varying concentrations of ERG to designated experimental groups. As depicted in Figure 4A–C, the control group exhibited normal skin color and smooth texture, demonstrating adequate water retention capacity and intact epidermal stratification with discernible cellular structures. Quantitatively, the trans-epidermal water loss (TEWL) was 12.27 ± 0.83 g/m^2^/h, and the epidermal thickness was 66.79 ± 14.73 μm. Conversely, the UVB-only model group developed typical photodamage symptoms including erythema, desquamation, and erosion, accompanied by pathological epidermal thickening (224.67 ± 20.52 μm) with disordered keratinocyte differentiation and a significantly elevated TEWL of 72.97 ± 1.96 g/m^2^/h. ERG treatment effectively alleviated these photodamage manifestations, restoring skin texture and histological morphology closer to a normal physiological status. Specifically, compared with the UVB-only model group, the ERG (200 mg/kg) group exhibited significant reductions in TEWL (48.34 ± 7.81 g/m^2^/h) and epidermal thickness (132.59 ± 27.97 μm), demonstrating therapeutic efficacy comparable to that of VE.

**Figure 5:**
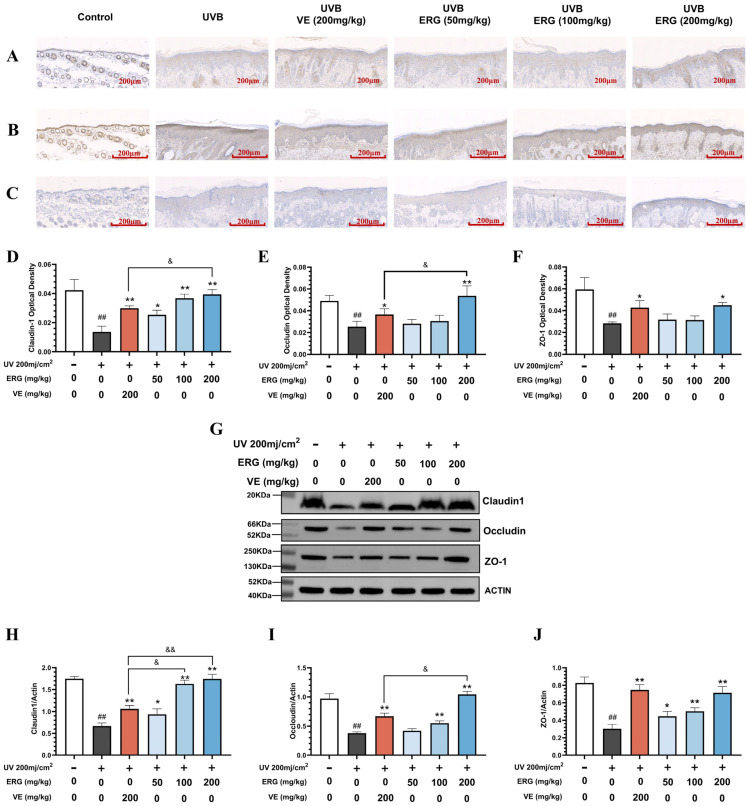
ERG restores key tight junction proteins in the epidermis of UVB-damaged mice. Skin tissue was collected from the experimental groups described in Figure 5. The expression and localization of key tight junction proteins were assessed by immunohistochemistry (IHC) and Western blot. (**A**–**C**) Representative IHC images showing the distribution of (**A**) Claudin-1, (**B**) Occludin, and (**C**) ZO-1 in the epidermis. Scale bar: 200 μm (**D**–**F**) Densitometric quantification of the IHC staining for (**A**) Claudin-1, (**B**) Occludin, and (**C**) ZO-1 (**G**) Representative Western blot analysis of Claudin-1, Occludin, and ZO-1 protein levels in total skin lysates. (**H**–**J**) Densitometric quantification of the Western blot results for (**H**) Claudin-1, (**I**) Occludin, and (**J**) ZO-1, normalized to a loading control (β-actin). Data in (**H**–**J**) are presented as the mean ± SD (*n* = 6). Statistical significance was determined by one-way ANOVA with Tukey’s post hoc test. ## *p* < 0.01 compared with the control group; * *p* < 0.05 and ** *p* < 0.01 compared with the UVB-only model group. & *p* < 0.05 and && *p* < 0.01 compared with the VE group.

To elucidate the molecular basis for this functional recovery, we examined key tight junction proteins. Immunohistochemical (IHC) analysis revealed that UVB irradiation severely disrupted the expression and epidermal localization of Claudin-1, Occludin, and ZO-1 (Figure 5A–C). ERG treatment significantly attenuated the UV-induced degradation of these proteins and largely restored their normal expression levels. Western blot analysis (Figure 5D–G) further corroborated these findings, demonstrating significantly elevated levels of all three junction proteins following ERG intervention, which was consistent with the IHC results.

### 2.5. ERG Suppresses UVB-Induced Skin Inflammation in Mice by Inhibiting the NF-κB Pathway

The observed improvements in skin morphology and barrier function suggest that ERG effectively counteracts the pathological effects of UVB exposure. Given that inflammation is a primary driver of such damage, we next investigated whether ERG’s therapeutic effect involved the suppression of the inflammatory cascade by analyzing the expression of key pro-inflammatory cytokines in mouse skin tissue. ELISA was employed to detect the levels of these cytokines.

**Figure 6:**
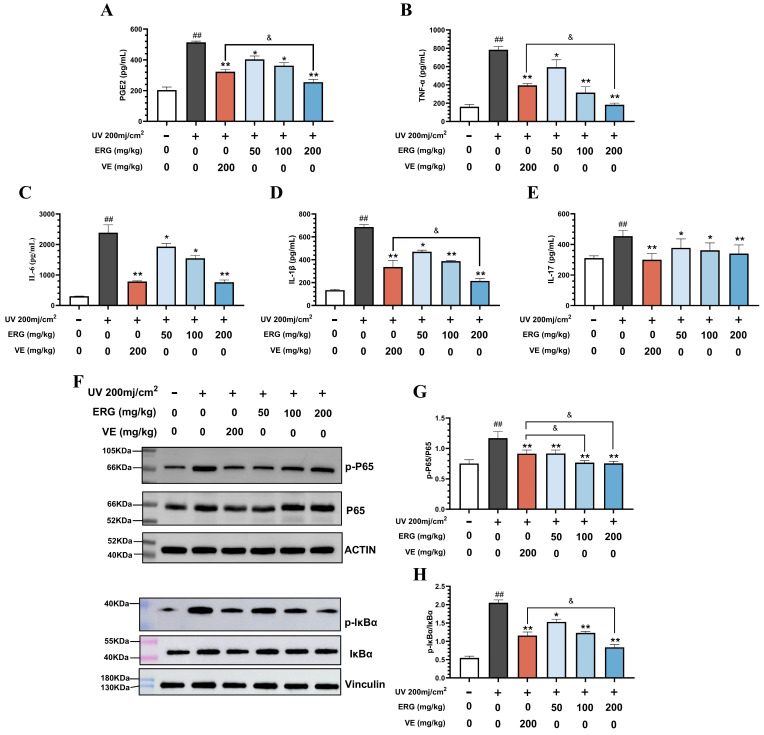
ERG alleviates UVB-induced skin inflammation via inhibition of the NF-κB pathway in mice. Skin tissue lysates from the different experimental groups were analyzed for cytokine levels and key signaling proteins. (**A**) ELISA quantification of prostaglandin E2 (PGE2). (B-E) ELISA quantification of pro-inflammatory cytokines: (**B**) TNF-α, (**C**) IL-6, (**D**) IL-1β, and (**E**) IL-17. (**F**) Representative Western blot images showing levels of phosphorylated IκBα (p-IκBα), phosphorylated p65 (p-p65), total p65, phosphorylated IκBα (p-IκBα), total IκBα, and loading controls (β-actin and Vinculin). (**G**,**H**) Densitometric quantification of the Western blot results for (**G**) p-P65/P65 and (**H**) p-IκB/ IκB. Data are presented as the mean ± SD (*n* = 6). Statistical significance was determined by one-way ANOVA with Tukey’s post hoc test. ## *p* < 0.01 compared with the control group; * *p* < 0.05 and ** *p* < 0.01 compared with the UVB-only model group. & *p* < 0.05 compared with the VE group.

Consistent with the observed tissue repair, ERG treatment potently suppressed the inflammatory environment in UVB-exposed skin. Analysis of skin tissue by ELISA confirmed that UVB irradiation triggered a significant elevation in key pro-inflammatory cytokines, an effect that was strongly reversed by ERG in a dose-dependent manner (Figure 6A–E). We first examined prostaglandin E2 (PGE2), a key lipid mediator of inflammation. UVB irradiation significantly elevated PGE2 levels, and this elevation was markedly suppressed by ERG treatment (Figure 6A). Notably, the inhibition achieved by ERG (200 mg/kg) was 50.36% greater than that of VE (37.09%) The favorable anti-inflammatory activity of ERG was further corroborated in the expression of cytokine mediators. Specifically, UVB stimulation caused an approximate 5-fold surge in TNF-α levels compared to the control group (*p* < 0.01). While the positive control VE significantly reduced TNF-α by 49.63%, ERG treatment demonstrated a superior inhibitory effect, with the high-dose group (ERG, 200 mg/kg) achieving a 76.61% reduction (Figure 6B). A similar pattern was observed for IL-1β, which was markedly elevated in the UVB-only model group (*p* < 0.01). Here again, ERG (200 mg/kg, 68.64% inhibition) outperformed VE (50.88% inhibition) in suppressing IL-1β levels (Figure 6C). Both ERG and VE also strongly counteracted the UVB-induced increase in IL-6. The inhibitory effect of ERG (200 mg/kg) was highly significant (*p* < 0.01), achieving a 68.11% reduction that was comparable to the potent effect of VE (67.17% reduction) (Figure 6D). Finally, all treatments significantly lowered IL-17 expression, with VE showing the greatest effect (34.12% inhibition; *p* < 0.01). ERG also dose-dependently suppressed IL-17, achieving a 25.19% reduction at the highest concentration (*p* < 0.05) (Figure 6E).

To further elucidate the anti-inflammatory mechanism of ERG in UVB-induced cutaneous photodamage in mice, we performed Western blot analysis to assess the phosphorylation levels of IκBα and P65, key components of the NF-κB signaling pathway. As illustrated in Figure 6F, UVB irradiation significantly elevated the phosphorylation levels of both IκBα and P65 in the dorsal skin of mice. However, following treatment with ERG, these phosphorylation levels were significantly reduced compared to those in the UVB-only model group. Notably, the inhibitory effects on P65 phosphorylation in both the medium-dose and ERG (200 mg/kg) groups were more pronounced than those observed in the Control group. Specifically, P65 phosphorylation was reduced by 1.52-fold in the ERG (100 mg/kg) group and 1.55-fold in the ERG (200 mg/kg) group, compared to the UVB-only model group, whereas the VE group showed a 1.28-fold reduction (Figure 6G). Furthermore, in the ERG (200 mg/kg) group, the phosphorylation of IκBα was reduced by 2.45-fold compared to the UVB-only model group (Figure 6H). These findings collectively suggest that ERG exerts its anti-inflammatory effects by suppressing the activation of the NF-κB pathway through the inhibition of IκBα and P65 phosphorylation, thereby downregulating the expression of pro-inflammatory cytokines such as TNF-α, IL-6, IL-1β, and IL-17.

### 2.6. ERG Proctects the Dermal Collagen Matrix in Mouse Skin by Suppressing the MAPK Pathway

In addition to the acute inflammatory response, chronic UVB exposure is known to lead to photoaging, primarily characterized by the degradation of the dermal collagen matrix. This destructive process is largely governed by the activation of the MAPK signaling pathway and the subsequent overexpression of MMPs [7]. Therefore, we next sought to determine if ERG could also protect against UVB-induced damage to the skin’s collagen framework.

**Figure 7:**
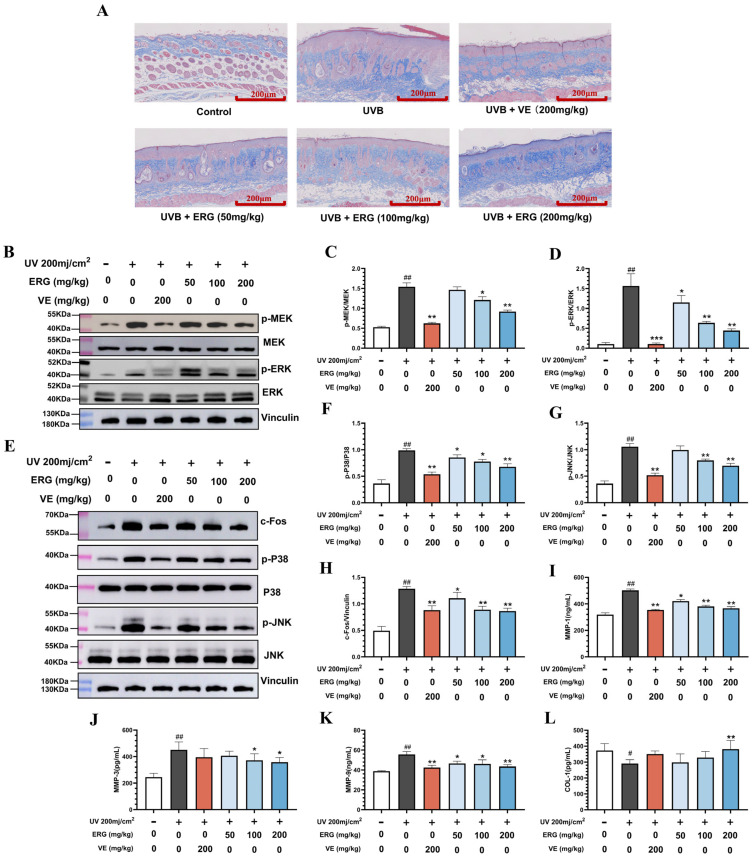
ERG Protects the dermal collagen matrix from UVB-induced degradation by inhibiting the MAPK/ERK/MMP pathway. Skin tissue from the different experimental groups was analyzed for collagen integrity and key protein expression. (**A**) Representative Masson’s trichrome staining of dorsal skin sections (collagen is stained blue). (**B**) Representative Western blot images for phosphorylated MEK (p-MEK), total MEK, phosphorylated ERK (p-ERK) and total ERK. (**C**,**D**) Densitometric quantification of Western blot results: (**C**) p-MEK/MEK and (**D**) p-ERK/ERK. (**E**) Representative Western blot images for phosphorylated P38 (p-P38), total p38 phosphorylated JNK (p-JNK), total JNK and c-Fos (**F**–**H**) Densitometric quantification of Western blot results: (**F**) p-P38/P38 and (**G**) p-JNK/JNK and (**H**) c-Fos(I-K) ELISA quantification of matrix metalloproteinase (MMP) levels in skin tissue: (**I**) MMP-1, (**J**) MMP-3, and (**K**) MMP-9. (**L**) ELISA quantification of Collagen I content. Data are presented as the mean ± SD (*n* = 6). Statistical significance was determined by one-way ANOVA with Tukey’s post hoc test. # *p* < 0.05 and ## *p* < 0.01 compared with the control group; * *p* < 0.05, ** *p* < 0.01 and *** *p* < 0.001 compared with the UVB-only model group.

The alterations in collagen fibers within mouse dorsal skin were assessed using Masson staining, with the results presented in Figure 7A. Compared to healthy control mice, the collagen fibers in the skin of UVB-exposed mice exhibited disordered arrangement and significantly reduced content. Following ERG intervention, a notable increase in collagen fiber content and structural remodeling were observed. Given that ultraviolet light induces extracellular signal-regulated kinase (ERK), P38, and c-Jun N-terminal kinase (JNK) phosphorylation, which leads to elevated MMP expression and consequently accelerates collagen degradation while damaging the skin’s collagen matrix, we first investigated the inhibitory effect of ERG on the UVB-induced MAPK signaling pathway through Western blot analysis. As shown in Figure 7B–D, UVB exposure significantly increased MEK and ERK phosphorylation levels in the dorsal skin of mice. Following ERG treatment, the phosphorylation level of MEK decreased by 1.68-fold, and the phosphorylation level of ERK decreased by 3.54-fold compared to the UVB-only model group. We further examined ERG’s effects on two other key MAPK pathways: p38 and JNK. Western blot analysis revealed that UVB irradiation also significantly activated both p38 and JNK pathways. Importantly, ERG treatment effectively suppressed the phosphorylation of both p38 and JNK in a dose-dependent manner (Figure 7E–G). Specifically, the high-dose ERG (200 mg/kg) reduced p38 phosphorylation by 1.46-fold and JNK phosphorylation by 1.52-fold compared to the UVB-only group.

Concurrently, ERG treatment significantly reduced the expression level of c-Fos in the skin of mice (Figure 7H). As a component of the AP-1 transcription factor, c-Fos can directly activate the transcription of MMPs. Consequently, the expression levels of MMP-1, MMP-3, and MMP-9, and the content of collagen I in the mouse skin tissue were further examined by ELISA. After ERG treatment, the expression levels of MMP-1, MMP-3, and MMP-9 in the mouse skin decreased, and the content of collagen I increased (Figure 7I–L), which was consistent with the Masson staining results.

### 2.7. Transcriptomic Analysis Reveals ERG’s Global Regulatory Effects on UVB-Induced Skin Damage

**Figure 8:**
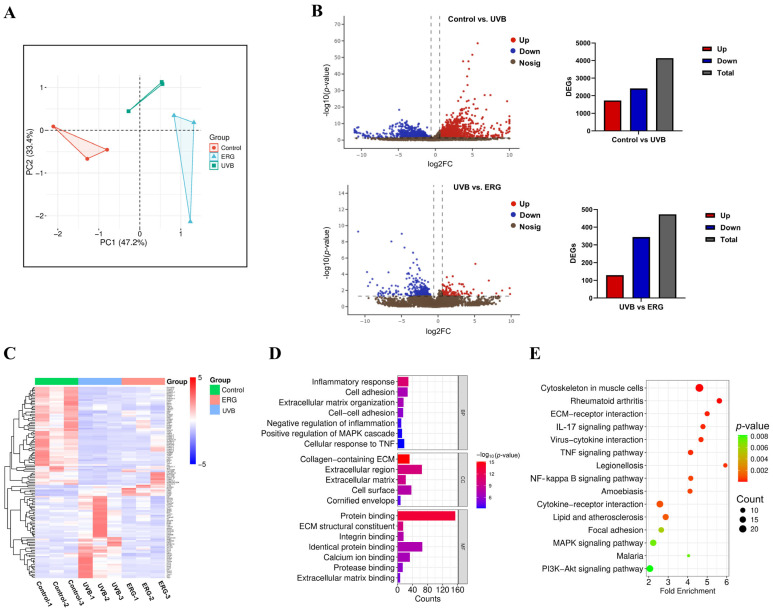
Transcriptomic analysis reveals ERG reverses UVB-induced inflammatory and matrix-remodeling gene signatures. RNA sequencing was performed on dorsal skin tissue from three experimental groups: Control, UVB-Model, and ERG (200 mg/kg) treated. Differentially expressed genes (DEGs) were identified with a significance threshold of *p* < 0.05 and |log2(Fold Change)| > 0.585. (**A**) PCA showing distinct clustering of the three experimental groups based on their global gene expression profiles. (**B**) Volcano plot illustrating DEGs between the ERG-treated group and the UVB-Model group. Significantly downregulated genes in the ERG group are shown in red, and upregulated genes are shown in cyan. (**C**) Clustering heatmap of DEGs identified between the ERG and UVB-Model groups. Red indicates higher relative expression, and blue indicates lower expression, showing that ERG treatment reverses many UVB-induced changes. (**D**) Gene Ontology (GO) enrichment analysis of DEGs, showing top terms for biological processes, cellular components, and molecular functions. (**E**) Kyoto Encyclopedia of Genes and Genomes (KEGG) pathway enrichment analysis of DEGs, highlighting the significant enrichment of the NF-κB and MAPK signaling pathways.

Our preceding molecular analyses indicated that ERG’s protective effects against UVB-induced skin damage are mediated, in part, by the inhibition of the NF-κB and MAPK/ERK signaling pathways. To comprehensively and unbiasedly investigate the global genetic reprogramming induced by ERG in response to UVB damage, and to further validate the involvement of these key pathways, we performed transcriptomic analysis on dorsal skin samples from the control, UVB-irradiated (model), and high-dose ERG-treated mice (Figure 8).

Differentially expressed genes (DEGs) were identified using DESeq2 for inter-group comparisons, with the screening threshold set as |log2(Fold Change)| > 0.585 (equivalent to FC = 1.5) and *p* < 0.05. Principal component analysis (PCA) (Figure 8A) illustrated the distinct transcriptional profiles and relationships between samples from different groups. Volcano plots (Figure 8B) visualized the differentially upregulated and downregulated genes. A total of 473 DEGs were detected between the ERG-treated group and the UVB-only model group, comprising 129 significantly upregulated genes and 344 significantly downregulated genes. Additionally, a hierarchical clustering heatmap (Figure 8C) provided a visual representation of the overall gene expression patterns. Specifically, following ERG intervention, the expression levels of several prominent pro-inflammatory genes, including tumor necrosis factor superfamily member 11 (*Tnfsf11*) and its receptor (*Tnfrsf1b*), interleukin 1 receptor-like 1 (*Il1rl1*), and CXC chemokines (*Cxcl1*, *Cxcl2*), were significantly downregulated compared to the UVB-only model group. This further corroborated the observed reduction in inflammatory cytokines.

To gain deeper insight into the biological functions and pathways affected by ERG, Gene Ontology (GO) and Kyoto Encyclopedia of Genes and Genomes (KEGG) pathway enrichment analyses were performed on the identified DEGs between the UVB-only model and ERG-treated groups. The GO analysis results (Figure 8D) revealed significant enrichment of biological processes related to inflammatory response, cell adhesion, and extracellular matrix organization. Enriched cellular components primarily included the extracellular matrix and plasma membrane, while molecular functions predominantly involved protein binding, integrin binding, and calcium ion binding. KEGG pathway enrichment analysis (Figure 8E) further highlighted that the primary regulatory mechanisms underlying ERG-mediated mitigation of skin photodamage involved the IL-17 signaling pathway, TNF signaling pathway, NF-κB signaling pathway, and MAPK signaling pathway.

It is well-established that UV radiation activates the NF-κB signaling pathway, often by inducing ROS production and DNA damage, which subsequently stimulates keratinocytes to release pro-inflammatory cytokines. These cytokines, like TNF, can further activate NF-κB through the TNFR1-RIP1-IKK cascade, forming a positive feedback loop that amplifies inflammation [31]. Our findings suggest that ERG intervenes in this cascade. Similarly, the MAPK signaling pathway, crucial for regulating collagen synthesis via MEK-mediated ERK phosphorylation, was implicated. The involvement of the IL-17 pathway also suggests ERG’s role in mitigating barrier compromise that can occur in inflammatory conditions [32].

Collectively, these transcriptomic insights corroborate our previous targeted findings and strongly suggest that ERG exerts its protective effects by comprehensively modulating inflammatory responses, maintaining skin barrier integrity, and influencing collagen matrix homeostasis, primarily through the regulation of the NF-κB, MAPK, and IL-17 signaling pathways.

### 2.8. Network Pharmacological Analysis Reinforces ERG’s Multi-Target Mechanisms

The transcriptomic data strongly supported our preceding experimental findings, further confirming that ERG comprehensively modulates gene networks related to inflammation and extracellular matrix remodeling. To further solidify these mechanistic insights from a complementary, predictive standpoint, we employed a network pharmacology analysis. The aim was to identify potential protein targets of ERG and map their intersection with known pathological pathways involved in skin photodamage.

**Figure 9:**
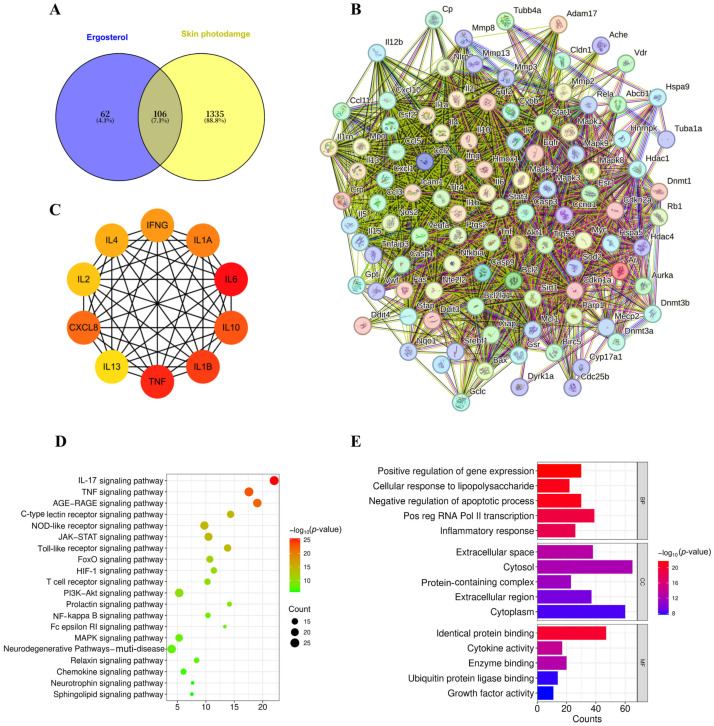
Network pharmacology predicts key targets and pathways for ergosterol in skin photodamage. Bioinformatic analysis was performed on 106 targets identified at the intersection of predicted ERG targets and known skin photodamage-associated genes. (**A**) Venn diagram showing the 106 common targets. (**B**) PPI network of the common targets (string cut-off value: 0.7). (**C**) A subnetwork highlighting the interactions between the top ten identified hub genes. (**D**) GO enrichment analysis showing the top ten enriched terms across the three main categories. (**E**) KEGG pathway analysis showing the top 20 significantly enriched signaling pathways. All enrichment analyses were performed with a significance threshold of *p* < 0.05.

First, potential targets of ERG were predicted using the SwissTargetPrediction and Comparative Toxicogenomics Database (CTD). Concurrently, skin photodamage-related targets were retrieved from the GeneCards database. Intersection analysis of these two target sets identified 106 common targets, visually represented in the Venn diagram (Figure 9A). These common targets were subsequently imported into the STRING database to construct a protein–protein interaction (PPI) network with a confidence score cut-off value of 0.7 (Figure 9B). Following this, Cytoscape software (Version 3.7.1) was utilized to analyze the PPI network topology, leading to the identification of the top ten key targets (hub genes) based on their highest node degrees.

Remarkably, among these key identified targets were critical inflammatory mediators such as IL-1β, TNF, and IL-6, whose expression levels were experimentally confirmed to be significantly reduced following ERG intervention (as shown in Section 2.5). Furthermore, the network analysis highlighted several other cytokines, including Interleukin-4 (IL-4), Interferon-γ (IFNG), Interleukin-1α (IL1A), Interleukin-2 (IL-2), C-X-C Motif Chemokine Ligand 8 (CXCL8), Interleukin-10 (IL-10), Interleukin-13 (IL-13), and Interleukin-18 (IL-18), as potential therapeutic targets or key nodes within the photodamage-associated network (Figure 9C). This network pharmacological approach thus provided predictive validation for our experimental observations and expanded the landscape of potential protein targets through which ERG exerts its protective effects against UVB-induced skin damage.

To further elucidate the biological functions and pathways associated with these 106 common targets, GO enrichment analysis was performed using the DAVID with a significance threshold of *p* < 0.05. This analysis yielded a comprehensive set of 493 enriched biological processes, 35 cellular components, and 86 molecular functions. The top ten enriched terms for each category are visually presented in Figure 9D. Specifically, biological processes were notably associated with cellular responses to lipopolysaccharides, inflammatory responses, and immune system processes. Enriched cellular components predominantly included the extracellular space, cytosol, extracellular region, and protein-containing complexes. Molecular functions were mainly concentrated on identical protein binding, cytokine activity, and enzyme binding.

Concurrently, KEGG pathway enrichment analysis was conducted (Figure 9E). This analysis identified 39 significantly enriched signaling pathways (*p* < 0.05), with the top 20 pathways visualized in ascending order of their *p*-values in Figure 9E. These findings from the network pharmacological enrichment analyses strongly converge with our experimental observations (Section 2.5, Section 2.6 and Section 2.7), suggesting that ERG may exert its protective effects against skin photodamage by comprehensively modulating critical signaling pathways such as IL-17 signaling pathway, TNF signaling pathway, NF-κB signaling pathway, and MAPK signaling pathway. This multi-pathway modulation appears to lead to a reduction in the release of inflammatory factors and the preservation of the collagen matrix, ultimately contributing to ERG’s anti-photoaging efficacy.

## 3. Discussion

**Figure 10:**
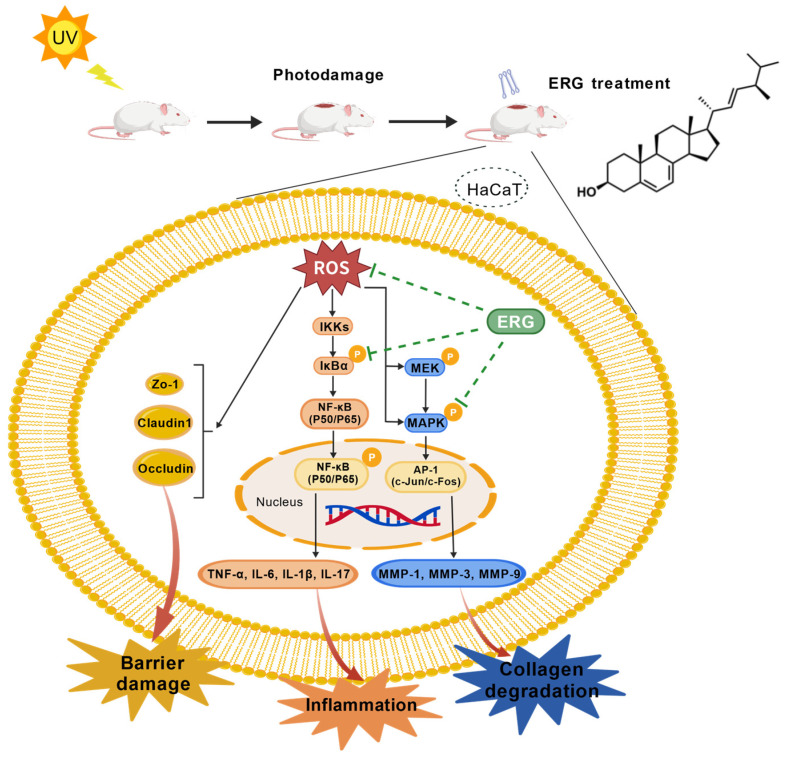
Schematic illustration of the proposed protective mechanism of ERG against UV-induced skin photodamage. UVB radiation generates excessive ROS, which activates two key pathological cascades. The pro-inflammatory NF-κB pathway drives cytokine release, leading to inflammation and skin barrier disruption. Concurrently, the MAPK pathway upregulates MMPs, causing degradation of the dermal collagen matrix. ERG exerts its comprehensive protection by quenching upstream ROS and inhibiting the MAPK signaling cascade. This dual action simultaneously suppresses inflammation, preserves skin barrier integrity, and prevents collagen degradation.

In this study, we demonstrate for the first time that ergosterol (ERG), a natural precursor to vitamin D2, provides robust protection against UVB-induced skin photodamage in both cellular and animal models. Our findings reveal that ERG operates through a powerful dual-action mechanism: it simultaneously suppresses the NF-κB-driven inflammatory cascade and inhibits the MAPK pathway responsible for collagen degradation (Figure 10). This multi-pronged approach allows ERG to effectively counteract both the acute inflammatory and chronic photoaging aspects of sun damage, positioning it as a promising candidate for dermatological applications. The observed effects may, in part, stem from ERG’s unique chemical structure, which functionally resembles cholesterol. This structural similarity allows ERG to integrate into lipid bilayers, potentially stabilizing membrane architecture by increasing lipid order and thickness [33]. This enhanced structural integrity reduces the membrane’s susceptibility to deformation and limits the ability of ROS to permeate it and initiate lipid peroxidation. By suppressing this peroxidation cascade, the formation of pro-inflammatory lipid-derived mediators is diminished, thereby mitigating the activation of subsequent inflammatory pathways [34].

ERG’s inhibitory effects may involve modulation of the NF-κB/Nrf2 signaling axis. A well-established reciprocal antagonism exists between these pathways: NF-κB-driven chronic inflammation sustains a state of oxidative stress while concurrently suppressing Nrf2-mediated antioxidant defenses [35]. Our data demonstrate that ERG effectively attenuates NF-κB activation and may restore redox homeostasis by counteracting the suppression of Nrf2. This dual-targeting mechanism—simultaneously dampening inflammatory signaling and potentiating the antioxidant response—provides a novel rationale for ERG’s efficacy against photodamage and merits further investigation.

UVB exposure induces a spectrum of dermatological pathologies, including erythema, dryness, desquamation, and hyperpigmentation, and may also lead to severe conditions such as melanoma, a potentially fatal form of skin cancer. Mechanistically, this occurs through the accumulation of ROS, which triggers oxidative stress and the subsequent upregulation of pro-inflammatory cytokines such as IL-1β and IL-6. This cascade of events disrupts the normal functions of keratinocytes, ultimately leading to extracellular matrix degradation, compromised skin barrier integrity, and impaired skin hydration [36,37,38,39]. Furthermore, ROS induced by UVB exposure may further impair autophagy response, potentially causing DNA damage and dysregulating autophagy processes, ultimately contributing to melanoma development [40]. A primary trigger of photodamage is oxidative stress, and our results show ERG potently quenches UVB-induced ROS in keratinocytes. This initial antioxidant effect is critical, as ROS is a known activator of the NF-κB pathway [41]. In our mouse UVB model, we confirmed that ERG treatment significantly inhibited NF-κB activation by preventing the phosphorylation of its key subunits, p65 and IκBα [42,43,44,45,46]. This mechanistic action directly explains the dramatic reduction we observed in downstream pro-inflammatory cytokines, such as IL-1β, IL-6, and TNF-α [47,48]. For instance, high-dose ERG suppressed TNF-α and IL-1β expression by 76.61% and 68.64%, respectively—an effect superior to the positive control VE. This potent anti-inflammatory activity, consistent with reports on other natural products like curcumin and proanthocyanidins, logically underlies the observed clinical improvements. By suppressing this inflammatory cascade, ERG was able to alleviate erythema and restore the integrity of the skin’s physical barrier, evidenced by the restored expression of key tight junction proteins Claudin-1, Occludin, and ZO-1 [49,50,51].

Beyond inflammation, ERG also directly addressed the structural damage associated with photoaging. We demonstrated that ERG treatment potently inhibited the UVB-induced phosphorylation of MEK and ERK, the central kinases in the MAPK pathway, by 1.68-fold and 3.54-fold, respectively. This blockade prevented the downstream expression of the transcription factor c-Fos (a key component of AP-1) and, consequently, suppressed the production of a panel of matrix-degrading enzymes including MMP-1, MMP-3, and MMP-9 [52]. The direct result of inhibiting these collagenases was the significant preservation and restoration of the Collagen I matrix, as confirmed by both Masson staining and ELISA. This demonstrates that ERG’s therapeutic action is not limited to controlling inflammation but also includes actively protecting the skin’s structural framework.

The centrality of these two pathways was powerfully corroborated by our unbiased transcriptomic analysis. KEGG enrichment confirmed that the IL-17, TNF, NF-κB, and MAPK signaling pathways were the most significantly modulated networks following ERG treatment. The synergistic feedback loop between IL-17 and TNF, as characterized by Li et al. [53], operates through a two-phase mechanism. The initial phase involves TNF-driven NF-κB activation, which induces the transcription of unstable inflammatory mRNAs. In the second phase, IL-17 signaling—dependent on the adaptor Act1 and RNA-binding proteins—post-transcriptionally stabilizes these mRNAs and increases their translation. This cooperation results in a significant amplification of the inflammatory response, establishing a feedforward circuit that is further cemented by secondary factors like IκBζ.

ERG’s ability to suppress genes across these interconnected pathways suggests it intervenes at multiple targets to disrupt this synergy. This provides a compelling explanation for the mechanistic distinction we observed between ERG and Vitamin E. While both were effective *in vivo*, VE’s failure in the direct LPS-macrophage model suggests its action is primarily ROS-scavenging, whereas ERG appears to be a more sophisticated modulator of specific inflammatory signaling networks. Furthermore, the downregulation of specific genes like *Tnsf11*, *Il1b*, and chemokines *Cxcl1/2* provided global, unbiased confirmation of our targeted findings. Interestingly, our results also highlighted a mechanistic distinction between ERG and VE. While both were effective *in vivo*, VE failed to suppress inflammation in the LPS-stimulated macrophage model. This divergence may stem from VE’s primary role as a ROS scavenger [54,55], which may be insufficient against the direct TLR4-mediated signaling induced by LPS [56,57]. ERG’s success in both models suggests its mechanism may be broader, potentially involving direct modulation of inflammatory receptors or signaling proteins.

While our findings robustly demonstrate the therapeutic potential of ergosterol (ERG) in preclinical models, several limitations and critical translational considerations must be acknowledged to contextualize this work. A primary constraint is the reliance on a murine model; despite its established utility in photodamage research, the architectural and immunological differences between murine and human skin necessitate future validation in human skin explants or clinical trials. Mechanistically, although we have identified key signaling pathways modulated by ERG (NF-κB and MAPK), its precise initial molecular targets—such as potential interactions with membrane receptors, signaling complexes, or other upstream components—remain to be fully elucidated. From a translational perspective, the pharmacological profile of topical ERG requires rigorous evaluation. While ERG is considered a safe biomaterial with beneficial bioactivity and no known adverse clinical effects related to cholesterol [58], its inherent low oil-solubility and tendency to crystallize present significant formulation challenges for direct dermatological application. Consequently, key parameters including its dermal penetration efficiency, potential for local metabolism by skin microbiota, systemic absorption, and the safety profile of any resulting metabolites must be definitively addressed through dedicated toxicokinetic and pharmacokinetic studies before its development as a viable dermatological agent can be realized.

## 4. Materials and Methods

### 4.1. Materials

ERG (purity > 97%) and 3-(4,5-dimethylthiazol-2-yl)-2,5-diphenyltetrazolium bromide (MTT) were purchased from Macklin Biochemical Co., Ltd. (Shanghai, China). Dexamethasone (DEX; purity ≥ 98%) and Vitamin E (VE; purity > 97%) were obtained from Aladdin Biochemical Technology Co., Ltd. (Shanghai, China). Stock solutions for ERG, DEX, and VE were prepared in dimethyl sulfoxide (DMSO). Lipopolysaccharide (LPS), RIPA lysis buffer, and a nitric oxide (NO) assay kit were sourced from Beyotime Institute of Biotechnology (Haimen, China). Enzyme-linked immunosorbent assay (ELISA) kits for the quantification of IL-17, IL-1β, IL-6, TNF-α, PEG2, MMP-1, MMP-3, MMP-9, Collagen I (COL-I) and TNF-α were purchased from Elabscience Biotechnology Co., Ltd. (Wuhan, China). General laboratory reagents, including formaldehyde, ethanol, and xylene, were supplied by Sinopharm Chemical Reagent Co., Ltd. (Shanghai, China). Dulbecco’s modified Eagle medium (DMEM), penicillin-streptomycin solution, and a panel of primary antibodies against Occludin, Claudin-1, ZO-1, c-FOS, IκBα, phospho-IκBα, p65 and phospho-p65 (Ser536), were sourced from Servicebio Technology Co., Ltd. (Wuhan, China). Fetal bovine serum (FBS) and the HRP-conjugated goat anti-rabbit IgG secondary antibody were obtained from Wuhan Procell Biotechnology Co., Ltd. (Wuhan, China).

### 4.2. Cell Culture and Treatment

Cell lines (HaCaT cells and RAW264.7) were obtained from the National Biomedical Laboratory Cell Resource Bank (Beijing, China). HaCaT cells were placed in a complete medium containing 10% fetal bovine serum and 1% penicillin-streptomycin antibiotic. RAW264.7 was cultured in a complete medium containing 12% fetal bovine serum), and then cultured in an incubator at 37 °C and 5% CO_2_. The cells were seeded onto 96-well plates at a density of 1 × 10^5^ per well and cultured overnight. After removing the supernatant and covering the cells with PBS, the cells were irradiated with a dose of 200 mJ/cm^2^ of UVB with Crosslinking instrument (Shanghai Luyang Instruments Co., Ltd., Shanghai, China). Discard PBS. In the drug-treated group, add 100 μL of ERG at various concentrations (5, 10, or 20 μM) and VE (20 μM) to each well. In both the UVB-only model group and the control group, add an equal volume of complete culture medium and continue incubation for 24 h, and the cell viability was detected by MTT.

### 4.3. MTT Assay for Cell Viability

HaCaT cells were inoculated into 96-well plates at a density of 5 × 10^3^ per well. After 24 h of culture, the old culture medium was aspirated, and the cells were treated with different concentrations of ERG (5, 10, or 20 μM) or positive control drug VE (20 μM), 30 min after UVB irradiation if necessary. After 24 h, add the MTT solution and incubate in the incubator for 4 h. Remove the supernatant and add dimethyl sulfoxide. The absorbance value was measured at 570 nm using a microplate reader (Life Technologies, Carlsbad, CA, USA).

### 4.4. Measurement of Intracellular ROS Production

HaCaT cells were seeded in 6-well plates at a density of 1.5 × 10^5^ per well and cultured overnight until adherent. After UVB irradiation, the cells were treated with VE (20 μM) and different concentrations of ERG (5, 10, and 20 μM) for 6 h. Add diluted DCFH-DA (10 μM) to fully cover the cells and incubate them in a 37 °C incubator for 20 min. After removing the culture medium, add 1 mL of PBS and observe under a fluorescence microscope (Life Technologies, Carlsbad, CA, USA).

### 4.5. Nitric Oxide Determination

RAW264.7 cells were seeded in 96-well plates at a density of 1 × 10^5^ cells per well. After 24 h, the cells were treated with LPS) at a concentration of 1 μg/mL to induce inflammation. Subsequently, dexamethasone (DEX, 5 μM), VE (20 μM), and varying concentrations of ERG (5, 10, and 20 μM) were added, and the cells were incubated for an additional 24 h. After the treatment is completed, collect 100 µL of the cell culture supernatant from each well and mix it with an equal volume of Griess reagent. After incubation at room temperature for 10 min, the absorbance value at 560 nm was measured using a microplate reader. The content of Nitric oxide (NO) was determined by comparing with the standard curve of sodium nitrite.

### 4.6. Enzyme-Linked Immunosorbent Assay

Raw264.7 cells were seeded into plates at a density of 2 × 10^5^ cells per well and incubated overnight in a humidified incubator at 37 °C with 5% CO_2_. Following incubation, the cell culture supernatant was collected and centrifuged at 4 °C at 3000 rpm for 20 min. The resulting supernatant was then used for subsequent analysis.

For skin tissue processing, dorsal skin samples were collected from mice, cut into small fragments, and homogenized in RIPA lysis buffer. The homogenate was centrifuged at 4 °C at 12,000 rpm for 10 min, and the supernatant was collected for analysis. According to the manufacturer’s instructions, the concentrations of IL-6, IL-17, IL-1β, TNF-α, MMP3, MMP-1, MMP-9, and COL-1 in the supernatants were quantified using corresponding detection kits.

### 4.7. Animal Experiments

**Scheme 1:**
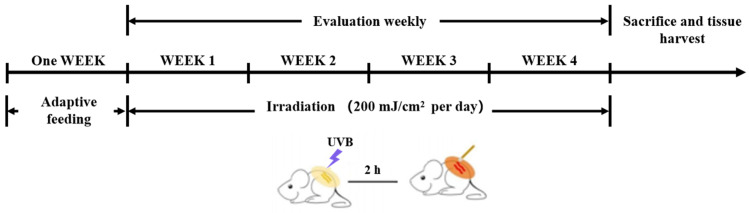
Schematic of the experimental protocol. The diagram illustrates the timeline for animal study, including the one-week acclimatization period, dorsal hair removal, and the daily 4-week cycle of UVB irradiation followed by topical application of PEG vehicle, VE, or ERG.

Female BALB/c mice (5–6 weeks old, 18–20 g) were procured from the Guangdong Medical Experimental Animal Center and housed under controlled conditions (23 ± 2 °C, 55 ± 5% humidity, 12 h light/dark cycle) with ad libitum access to chow and water. Both therapeutic agents, vitamin E (VE) and ERG, were suspended in PEG 400 as the vehicle at a concentration of 20 mg/mL. Following a one-week acclimatization period, the 36 mice were randomly assigned into six groups (*n* = 6 mice per group): a Control group (vehicle only, no UVB); a UVB Model group (vehicle and UVB exposure); a Positive Control (VE) group treated with 200 mg/kg topical VE; a group treated with 50 mg/kg topical ERG; a group treated with 100 mg/kg topical ERG; and a group treated with 200 mg/kg topical ERG. All groups, except for the Control, were subjected to UVB exposure following their respective treatments (Scheme 1).

Following the acclimatization period, dorsal hair was removed from all mice, first with an electric razor and subsequently with a commercial depilatory cream. A 48 h recovery period was permitted to prevent potential skin irritation from confounding the results. The experiment was conducted over four consecutive weeks. For the Model group and treatment groups (VE, 50 mg/kg ERG, 100 mg/kg ERG, 200 mg/kg ERG), mice were exposed to UVB radiation on the shaved dorsal skin. Two hours following each UVB exposure, the designated treatments—PEG vehicle, 200 mg/kg VE, or the respective doses of ERG (50, 100, 200 mg/kg)—were topically applied to the irradiated area. This irradiation-and-treatment cycle was repeated daily. The Control group received only the vehicle application without any hair removal or UVB exposure. A detailed schematic of the experimental timeline is presented in Scheme 1.

The experimental protocol was reviewed and approved by the Academic Integrity and Technology Ethics Committee of Guangdong University of Technology (Ethical Approval No. IACUC-AEWC-F241108001) on 10 Mar 2024, and conducted in full compliance with the National Research Council’s Guide for the Care and Use of Laboratory Animals (8th edition).

### 4.8. H&E Staining

The tissue samples were fixed in 10% neutral buffer formalin for 24 h, dehydrated with a gradient of ethanol, embedded in paraffin, and cut into sections with a thickness of 4–5 μm. The sections were placed on slides. After xylene dewaxing and gradient ethanol hydration, they were stained with hematoxylin and eosin and observed under a microscope.

### 4.9. Western Blot Analysis

The proteins of skin tissue samples were extracted using RIPA buffer containing protease inhibitors and quantified by the BCA method. The proteins were separated by SDS-PAGE gel electrophoresis and then transferred onto the PVDF membrane. The membrane was sealed with BSA solution and then incubated overnight with the primary antibodies of NF-κB p65, IκBα, p-IκBα, ERK, p-ERK, p-MEK, MEK, c-Fos, ZO-1, Occludin, Claudin-1 at 4 °C (dilution 1:500), and then incubated with the secondary antibody labeled horseradish peroxidase (HRP) at room temperature for 1 h. The protein bands were colored by chemiluminescence (ECL) reagents and quantitatively analyzed by comparison with internal reference protein.

### 4.10. Immunohistochemistry

Paraffin sections were dewaxed with xylene, gradually dehydrated with a gradient of anhydrous ethanol, and then treated with 3% hydrogen peroxide for 15 min. Dilute the primary antibodies (Claudin, Occludin, ZO-1) 100 times with distilled water and incubate overnight at 4 °C. Then add the secondary antibody (rabbit IgG enzyme-linked antibody), incubate at 37 °C for 50 min, and then stain with DAB and hematoxylin again. Random shooting and analysis were conducted at a magnification of 200 times.

### 4.11. Masson Staining

Skin tissues from the back of mice were taken, fixed and separated with 10% formalin, then embedded in paraffin and made into 4 μm sections. The sections were stained with hematoxylin, eosin and Masson tricolor, and histological analysis was performed under a microscope.

### 4.12. RNA-Seq and Transcriptome Analysis

Total RNA was extracted using Trizol reagent. Subsequently, mRNA was enriched using Oligo(dT) beads and fragmented to a size range of 200–700 nucleotides. The cDNA library was prepared employing the NEBNext Ultra RNA Library Prep Kit. Following adapter ligation and rolling circle amplification, paired-end sequencing (150 bp) was conducted on the Illumina NovaSeq 6000 platform. Raw reads were processed with Fastp to filter out low-quality sequences (Q-score ≤ 20) and remove adapter contamination. Ribosomal RNA sequences were aligned against the rRNA database using Bowtie2 (version 2.2.8), and the resulting clean reads were mapped to the reference genome via HISAT2 (version 2.4). Gene expression levels were quantified as FPKM (Fragments Per Kilobase of transcript per Million mapped reads) using StringTie (version 1.3.1), and differentially expressed genes (DEGs) were identified using DESeq2 with the criteria of *p* < 0.05 and |fold change| ≥ 1.5. Functional enrichment analysis (GO/KEGG) and alternative splicing detection (rMATS, *p* < 0.05) were subsequently performed.

### 4.13. Network Pharmacological Analysis

In the SwissTargetPrediction database (http://swisstargetprediction.ch/ (accessed on 23 June 2025) and CTD (https://ctdbase.org/, accessed on 23 June 2025), potential targets of ERG were collected. The collected target UniProt IDs were then imported into UniProt (https://www.uniprot.org/) for ID mapping, converting them into standardized gene names. In parallel, skin damage-related targets were gathered from GeneCards (https://www.genecards.org/, Version 5.24). Venny (https://www.bioinformatics.com.cn/static/others/jvenn/example.html, accessed on 23 June 2025, Version 2.1) was used to identify the overlapping targets between the predicted ERG targets and skin damage-associated targets. These intersecting targets were subsequently input into STRING (https://cn.string-db.org/, Version: 12.0) to construct a protein–protein interaction network, which was visualized using Cytoscape (Version 3.7.1) to display the top ten targets. Functional enrichment analysis of these intersection targets, including KEGG pathway and GO term enrichment, was conducted using the DAVID database (https://davidbioinformatics.nih.gov/home.jsp, accessed on 23 June 2025, version 2021), followed by data visualization via bioinformatics tools (https://www.bioinformatics.com.cn/).

### 4.14. Statistical Analysis

The results were expressed as mean ± standard deviation (SD). Data analysis was performed using GraphPad Prism 10.1.2 software (GraphPad Software, San Diego, CA, USA). Multi-component comparisons were conducted, and statistically significant values were analyzed using one-way analysis of variance (ANOVA) and Dunnett’s post hoc test. *p* < 0.05 indicates statistical significance.

## 5. Conclusions

In summary, this study demonstrates that the marine-derived sterol ERG mitigates UVB-induced photodamage by alleviating oxidative stress and regulating critical redox-sensitive pathways. Specifically, ERG functions not only as a ROS scavenger but also as an inhibitor of the pro-inflammatory NF-κB cascade and the MAPK signaling network, thereby preserving skin barrier function and inhibiting collagen degradation. Although both ERG and vitamin E showed efficacy *in vivo*, ERG’s additional anti-inflammatory action in macrophages suggests a multifaceted mechanism that surpasses conventional antioxidants. Consequently, ERG emerges as a compelling candidate for further research into therapies for oxidative stress-mediated skin aging.

## Data Availability

The raw data supporting the conclusions of this article will be made available by the authors on request.

## Abbreviations

The following abbreviations are used in this manuscript:

ABTS	2,2′-azino-bis(3-ethylbenzothiazoline-6-sulfonate)
AGE	advanced glycation end product
AG	aminoguanidine
Akt	protein kinase B
BSA	bovine serum albumin
cAMP	cyclic adenosine monophosphate
CREB	cAMP response element-binding protein
Cu^2+^	copper ion
DMSO	dimethyl sulfoxide
DMEM	Dulbecco’s modified Eagle’s medium
DEGs	differentially expressed genes
DPPH	1,1-diphenyl-2-picrylhydrazyl
EGFR	epidermal growth factor receptor
ERK	extracellular signal-regulated kinase
FBS	fetal bovine serum
FDR	false discovery rate
FPKM	fragments per kilobase of transcript per million mapped reads
GAPDH	glyceraldehyde 3-phosphate dehydrogenase
GSH	glutathione
GM-CSF	granulocyte-macrophage colony-stimulating factor
GO	gene ontology
GSK-3β	glycogen synthase kinase-3 beta
IC50	half-maximal inhibitory concentration
IL-18	interleukin 18
IL-33	interleukin 33
JAK-STAT	Janus kinase-signal transducer and activator of transcription
JNK	c- Jun N-terminal kinase
KEGG	Kyoto Encyclopedia of Genes and Genomes
MAPK	mitogen-activated protein kinase
MC1R	melanocortin 1 receptor
MGO	methylglyoxal
MITF	microphthalmia-associated transcription factor
MMP	matrix metalloproteinase
mRNA	messenger RNA
MSH	melanocyte-stimulating hormone
MTT	3-(4,5-dimethylthiazol-2-yl)-2,5-diphenyl tetrazolium bromide
NF-κB	nuclear factor kappa-light-chain-enhancer of activated B cells
PBS	phosphate-buffered saline
PCA	principal component analysis
PGE2	prostaglandin E2
PI3K	phosphatidylinositol 3-kinase
PKA	protein kinase A
PMSF	phenylmethylsulphonyl fluoride
qPCR	quantitative polymerase chain reaction
RNA-seq	RNA sequencing
RNS	reactive nitrogen species
ROS	reactive oxygen species
SP	Spirulina peptides
TNF	tumor necrosis factor
TRP-1	tyrosinase-related protein 1
TRP-2	tyrosinase-related protein 2
TYR	tyrosinase
UV	ultraviolet
UVB	ultraviolet B

## References

[1] Pedic L., Pondeljak N., Situm M. (2020). Recent information on photoaging mechanisms and the preventive role of topical sunscreen products. Acta Dermatovenerol. Alp. Pannonica Et Adriat..

[2] Li Z., Jiang R., Liu J., Xu X., Sun L., Zhao D. (2021). *Panax ginseng* C. A. Meyer Phenolic Acid Extract Alleviates Ultraviolet B-Irradiation-Induced Photoaging in a Hairless Mouse Skin Photodamage Model. Evid.-Based Complement. Altern. Med. Ecam.

[3] Muhammad N., Sulong M.S., Bakar M.F.A., Latif Abu Bakar M.A., Mayzan M.Z.H., Rahim N.F.A., Rashidi W.N.A.S.W.M., Pauzi A.N., Hussin N.B., Ismail N.I.F.N. (2025). Electronic nose investigation and antioxidant assessment of CHARMS™ skincare cosmetics toward skin tone improvement. J. Dermatol. Sci. Cosmet. Technol..

[4] Lone A.N., Malik A.T., Naikoo H.S., Raghu R.S., Tasduq S.A. (2020). Trigonelline, a naturally occurring alkaloidal agent protects ultraviolet-B (UV-B) irradiation induced apoptotic cell death in human skin fibroblasts via attenuation of oxidative stress, restoration of cellular calcium homeostasis and prevention of endoplasmic reticulum (ER) stress. J. Photochem. Photobiol. B Biol..

[5] Zhang X.J., Yang P.Y., Ding L., Wang J., Li X.L., Xiao W.L. (2025). Isolicoflavonol alleviates UVB-induced photodamage via protecting mitochondria and blocking the activation of NLRP3 inflammasome. Toxicol. Appl. Pharmacol..

[6] Li J., Wang S., Han R., Wu M., Zhou J., Zhao P., Cui B. (2024). A cutting-edge atomization-based methodology for enhancing formulation ability to resist photoaging. J. Dermatol. Sci. Cosmet. Technol..

[7] Chen Q., Lin W., Tang Y., He F., Liang B., Chen J., Li H., Zhu H. (2025). Curcumin targets YAP1 to enhance mitochondrial function and autophagy, protecting against UVB-induced photodamage. Front. Immunol..

[8] Liu K., Zhao C., Zhang K., Yang X., Feng R., Zong Y., He Z., Zhao Y., Du R. (2024). Pilose Antler Protein Relieves UVB-Induced HaCaT Cells and Skin Damage. Molecules.

[9] Lu W., Kong C., Cheng S., Xu X., Zhang J. (2023). Succinoglycan riclin relieves UVB-induced skin injury with anti-oxidant and anti-inflammatory properties. Int. J. Biol. Macromol..

[10] Saitoh Y., Tanaka A., Hyodo S. (2021). Protective Effects of Polyvinylpyrrolidone-Wrapped Fullerene Against Nitric Oxide/Peroxynitrite-Induced Cellular Injury in Human Skin Keratinocytes. J. Nanosci. Nanotechnol..

[11] Hegedus C., Juhasz T., Fidrus E., Janka E.A., Juhasz G., Boros G., Paragh G., Uray K., Emri G., Remenyik E. (2021). Cyclobutane pyrimidine dimers from UVB exposure induce a hypermetabolic state in keratinocytes via mitochondrial oxidative stress. Redox Biol..

[12] Fidrus E., Hegedus C., Janka E.A., Paragh G., Emri G., Remenyik E. (2021). Inhibitors of Nucleotide Excision Repair Decrease UVB-Induced Mutagenesis-An In Vitro Study. Int. J. Mol. Sci..

[13] Togsverd-Bo K., Philipsen P.A., Haedersdal M., Wulf H.C.O. (2018). Skin autofluorescence reflects individual seasonal UV exposure, skin photodamage and skin cancer development in organ transplant recipients. J. Photochem. Photobiol. B Biol..

[14] Zhong J., Liang L., Zhao N., Wang J., Shu P. (2025). Synergistic effects of retinol and retinyl palmitate in alleviating UVB-induced DNA damage and promoting the homologous recombination repair in keratinocytes. Front. Pharmacol..

[15] Ma X.Y., Wang H.N., Sun L.X., Sun J., Jin S.H., Dai F.X., Sai C.M., Zhang Z. (2025). Bioactive steroids from marine-derived fungi: A review (2015–2023). J. Asian Nat. Prod. Res..

[16] Hannan M.A., Sohag A.A.M., Dash R., Haque M.N., Mohibbullah M., Oktaviani D.F., Hossain M.T., Choi H.J., Moon I.S. (2020). Phytosterols of marine algae: Insights into the potential health benefits and molecular pharmacology. Phytomedicine.

[17] Carreon-Palau L., Ozdemir N.S., Parrish C.C., Parzanini C. (2020). Sterol Composition of Sponges, Cnidarians, Arthropods, Mollusks, and Echinoderms from the Deep Northwest Atlantic: A Comparison with Shallow Coastal Gulf of Mexico. Mar. Drugs.

[18] Rangsinth P., Sharika R., Pattarachotanant N., Duangjan C., Wongwan C., Sillapachaiyaporn C., Nilkhet S., Wongsirojkul N., Prasansuklab A., Tencomnao T. (2023). Potential Beneficial Effects and Pharmacological Properties of Ergosterol, a Common Bioactive Compound in Edible Mushrooms. Foods.

[19] Tian X., Qin K., Deng Y., Xue P., Huang C., Liu S., Hu Z. (2025). Expression pattern, subcellular localization of Aspergillus oryzae ergosterol synthases, and their effects on ergosterol and fatty acid metabolism. Appl. Environ. Microbiol..

[20] Zhou X., Hilk A., Solis N.V., Pereira De Sa N., Hogan B.M., Bierbaum T.A., Del Poeta M., Filler S.G., Burrack L.S., Selmecki A. (2024). Erg251 has complex and pleiotropic effects on sterol composition, azole susceptibility, filamentation, and stress response phenotypes. PLoS Pathog..

[21] Dupont S., Fleurat-Lessard P., Cruz R.G., Lafarge C., Grangeteau C., Yahou F., Gerbeau-Pissot P., Abrahao Junior O., Gervais P., Simon-Plas F. (2021). Antioxidant Properties of Ergosterol and Its Role in Yeast Resistance to Oxidation. Antioxidants.

[22] Makitaipale J., Opsomer H., Steiner R., Riond B., Liesegang A., Clauss M., Hatt J.M. (2024). Serum vitamin D concentrations in rabbits (*Oryctolagus cuniculus*) are more affected by UVB irradiation of food than irradiation of animals. Vet. J. (Lond. Engl. 1997).

[23] Miraglia Del Giudice M., Indolfi C., Strisciuglio C. (2018). Vitamin D: Immunomodulatory Aspects. J. Clin. Gastroenterol..

[24] Sun P., Li W., Guo J., Peng Q., Ye X., Hu S., Liu Y., Liu W., Chen H., Qiao J. (2023). Ergosterol Isolated from Antrodia camphorata Suppresses LPS-Induced Neuroinflammatory Responses in Microglia Cells and ICR Mice. Molecules.

[25] Tada H., Kawahara K., Osawa H., Song L.T., Numazaki K., Kawai J., Onoue S., Nishioka T., Nemoto E., Matsushita K. (2022). Hericium erinaceus ethanol extract and ergosterol exert anti-inflammatory activities by neutralizing lipopolysaccharide-induced pro-inflammatory cytokine production in human monocytes. Biochem. Biophys. Res. Commun..

[26] Sillapachaiyaporn C., Mongkolpobsin K., Chuchawankul S., Tencomnao T., Baek S.J. (2022). Neuroprotective effects of ergosterol against TNF-alpha-induced HT-22 hippocampal cell injury. Biomed Pharmacother..

[27] Yan C.Y., Zhu Q.Q., Guan C.X., Xiong G.L., Chen X.X., Gong H.B., Li J.W., Ouyang S.H., Kurihara H., Li Y.F. (2024). Antioxidant and Anti-Inflammatory Properties of Hydrolyzed Royal Jelly Peptide in Human Dermal Fibroblasts: Implications for Skin Health and Care Applications. Bioengineering.

[28] Li C., Zhu Y., Liu W., Hayashi T., Xiang W., He S., Mizuno K., Hattori S., Fujisaki H., Ikejima T. (2023). Increased mitochondrial fission induces NLRP3/cGAS-STING mediated pro-inflammatory pathways and apoptosis in UVB-irradiated immortalized human keratinocyte HaCaT cells. Arch. Biochem. Biophys..

[29] Eom J.W., Lim J.W., Kim H. (2023). Lutein Induces Reactive Oxygen Species-Mediated Apoptosis in Gastric Cancer AGS Cells via NADPH Oxidase Activation. Molecules.

[30] Luo J., Li C., Zhu Y., Guo R., Huang J., Yu H., Sun M., Zhu Q., Guo Q., Li Y. (2025). Deficiency of inducible nitric oxide synthase (iNOS) enhances MC903-induced atopic dermatitis-like inflammation in mice. Biochem. Biophys. Res. Commun..

[31] Wei M., Li M., Li Y., Wang B., Yan Y., Li L. (2025). Upregulation of Receptor Interacting Protein 1 Induced by UVB Contributes to Photodamage of the Skin Through NF-kappaB Pathway In Vivo and In Vitro. J. Cosmet. Dermatol..

[32] Johnston L.A., Nagalla R.R., Li M., Whitley S.K. (2024). IL-17 Control of Cutaneous Immune Homeostasis. J. Investig. Dermatol..

[33] Qian S., Nagy G., Zolnierczuk P., Mamontov E., Standaert R. (2024). Nonstereotypical Distribution and Effect of Ergosterol in Lipid Membranes. J. Phys. Chem. Lett..

[34] Zheng Y., Sun J., Luo Z., Li Y., Huang Y. (2024). Emerging mechanisms of lipid peroxidation in regulated cell death and its physiological implications. Cell Death Dis..

[35] Zhu X., Zhao Q., Wang P., Cao S., Williams J.P., An J. (2025). Ozone rectal insufflation attenuates lung inflammation by inhibiting the TLR4/NF-kappaB pathway through upregulation of Nrf2 in mice with COPD-like pathology. Int. Immunopharmacol..

[36] Tang H., Xu C., Ge Y., Xu M., Wang L. (2023). Multiparametric Quantitative Analysis of Photodamage to Skin Using Optical Coherence Tomography. Sensors.

[37] Tong T., Geng R., Kang S.G., Li X., Huang K. (2024). Revitalizing Photoaging Skin through Eugenol in UVB-Exposed Hairless Mice: Mechanistic Insights from Integrated Multi-Omics. Antioxidants.

[38] Xu D., Zhao M., Lin H., Li C. (2022). Theragra chalcogramma Hydrolysates, Rich in Gly-Leu-Pro-Ser-Tyr-Thr, Exerts Anti-Photoaging Potential via Targeting MAPK and NF-kappaB Pathways in SD Rats. Mar. Drugs.

[39] Lin Z.C., Hsu C.Y., Hwang E., Wang P.W., Fang J.Y. (2023). The role of cytokines/chemokines in an aging skin immune microenvironment. Mech. Ageing Dev..

[40] Umar S.A., Tasduq S.A. (2025). Photophagy: Unveiling a Novel Cellular Mechanism in UVB-Induced Skin Aging and Resilience. Int. J. Dermatol..

[41] Fu X., Zhang Y., Chen G., Mao G., Tang J., Xu J., Han Y., Chen H., Ding L. (2025). Responsive nanoparticles synergize with Curcumin to break the “reactive oxygen Species-Neuroinflammation” vicious cycle, enhancing traumatic brain injury outcomes. J. Nanobiotechnology.

[42] Nie Y., Li Y. (2025). Curcumin: A potential anti-photoaging agent. Front. Pharmacol..

[43] Yi Y., Li T., Lv C., He W., Li W., Zhou X., Qin S. (2024). Proanthocyanidins isolated from lotus seed skin mitigate glycolipid metabolism disorder through the p38/Nrf2/NF-kappaB signaling pathway. Acta Biochim. Et Biophys. Sin..

[44] Lu Z., Xia Q., Cheng Y., Lu Q., Li Y., Zeng N., Luan X., Li Y., Fan L., Luo D. (2022). Hesperetin attenuates UVA-induced photodamage in human dermal fibroblast cells. J. Cosmet. Dermatol..

[45] Zhang X., Hu S., Luo P., Li Z., Chen Z., Xia C., Fan L., Li R., Chen H. (2025). The regulatory effect and molecular mechanism of Epstein-Barr virus protein LMP-1 in SLE susceptibility gene expression. Immunol. Lett..

[46] Sun X., Cao S., Mao C., Sun F., Zhang X., Song Y. (2024). Post-translational modifications of p65: State of the art. Front. Cell Dev. Biol..

[47] Li Z., Shang W., Mei T., Fu D., Xi F., Shao Y., Song X., Wang Z., Qi K., Tu J. (2024). Outer membrane vesicles of avian pathogenic Escherichia coli induce necroptosis and NF-kappaB activation in chicken macrophages via RIPK1 mediation. Res. Vet. Sci..

[48] Ji L., Shi X., Wang G., Wu H., Hu Z. (2023). Overexpressing six-transmembrane protein of prostate 2 (STAMP2) alleviates sepsis-induced acute lung injury probably by hindering M1 macrophage polarization via the NF-kappaB pathway. Folia Histochem. Et Cytobiol..

[49] Imafuku K., Iwata H., Natsuga K., Okumura M., Kobayashi Y., Kitahata H., Kubo A., Nagayama M., Ujiie H. (2023). Zonula occludens-1 distribution and barrier functions are affected by epithelial proliferation and turnover rates. Cell Prolif..

[50] Serra D., Garroni G., Cruciani S., Coradduzza D., Pashchenko A., Amler E., Pintore G., Parisse P., Satta R., Martini F. (2025). PVA and PVP nanofibers combined with Helichrysum italicum oil preserve skin cell interactions, elasticity and proliferation. Sci. Rep..

[51] Arnold K.A., Moran M.C., Shi H., van Vlijmen-Willems I., Rodijk-Olthuis D., Smits J.P.H., Brewer M.G. (2024). CLDN1 knock out keratinocytes as a model to investigate multiple skin disorders. Exp. Dermatol..

[52] Lee J.M., Park S.J., Kim Y.J., Kim S.Y., Jang Y.N., Park A.Y., Ho S.H., Kim D., Lee J.O., Yoo K.H. (2024). Actinidia polygama Water Extract (APWE) Protects Against UVB-Induced Photoaging via MAPK/AP-1 and TGFbeta-Smad Pathway. Ann. Dermatol..

[53] Li X., Bechara R., Zhao J., McGeachy M.J., Gaffen S.L. (2019). IL-17 receptor-based signaling and implications for disease. Nat. Immunol..

[54] Faizan M., Alam P., Rajput V.D., Shareen, Kaur K., Faraz A., Minkina T., Maqbool Ahmed S., Rajpal V.R., Hayat S. (2023). Potential role of tocopherol in protecting crop plants against abiotic stresses. Physiol. Mol. Biol. Plants Int. J. Funct. Plant Biol..

[55] Chen J., Tai M., Chen J., Ni J., Yi H., Chen L., Wang D., Wen C., Li J., Shen X. (2024). Panax ginseng extract prevents UVB-induced skin photodamage by modulating VMP1-mediated ER stress. Phytomedicine.

[56] Zhu Y., Han Q., Wang L., Wang B., Chen J., Cai B., Wu C., Zhu X., Liu F., Han D. (2023). Jinhua Qinggan granules attenuates acute lung injury by promotion of neutrophil apoptosis and inhibition of TLR4/MyD88/NF-kappaB pathway. J. Ethnopharmacol..

[57] Luo R., Yao Y., Chen Z., Sun X. (2025). An examination of the LPS-TLR4 immune response through the analysis of molecular structures and protein-protein interactions. Cell Commun. Signal. CCS.

[58] Park S.H., Kim H.K. (2020). Antibacterial activity of emulsions containing unsaturated fatty acid ergosterol esters synthesized by lipase-mediated transesterification. Enzym. Microb. Technol..

